# Guanylate-Binding Protein 1: An Emerging Target in Inflammation and Cancer

**DOI:** 10.3389/fimmu.2019.03139

**Published:** 2020-01-24

**Authors:** Alexander T. Honkala, Dhanir Tailor, Sanjay V. Malhotra

**Affiliations:** Department of Radiation Oncology, School of Medicine, Stanford University, Stanford, CA, United States

**Keywords:** guanylate-binding protein 1, cytokine-induced GTPase, inflammation, interferon α (IFN α), infection, cancer

## Abstract

Guanylate-binding protein 1 (GBP1) is a large GTPase of the dynamin superfamily involved in the regulation of membrane, cytoskeleton, and cell cycle progression dynamics. In many cell types, such as endothelial cells and monocytes, GBP1 expression is strongly provoked by interferon γ (IFNγ) and acts to restrain cellular proliferation in inflammatory contexts. In immunity, GBP1 activity is crucial for the maturation of autophagosomes infected by intracellular pathogens and the cellular response to pathogen-associated molecular patterns. In chronic inflammation, GBP1 activity inhibits endothelial cell proliferation even as it protects from IFNγ-induced apoptosis. A similar inhibition of proliferation has also been found in some tumor models, such as colorectal or prostate carcinoma mouse models. However, this activity appears to be context-dependent, as in other cancers, such as oral squamous cell carcinoma and ovarian cancer, GBP1 activity appears to anchor a complex, taxane chemotherapy resistance profile where its expression levels correlate with worsened prognosis in patients. This discrepancy in GBP1 function may be resolved by GBP1's involvement in the induction of a cellular senescence phenotype, wherein anti-proliferative signals coincide with potent resistance to apoptosis and set the stage for dysregulated proliferative mechanisms present in growing cancers to hijack GBP1 as a pro- chemotherapy treatment resistance (TXR) and pro-survival factor even in the face of continued cytotoxic treatment. While the structure of GBP1 has been extensively characterized, its roles in inflammation, TXR, senescence, and other biological functions remain under-investigated, although initial findings suggest that GBP1 is a compelling target for therapeutic intervention in a variety of conditions ranging from chronic inflammatory disorders to cancer.

## Introduction

In molecular biology, it is common to consider the proteins involved in a given phenotype in isolation from their spatiotemporal cellular context. With guanylate-binding protein 1 (GBP1), such isolation is impossible. GBP1 appears to interact with a variety of binding partners in multiple compartments, including extracellular signaling via secretion, endosomal trafficking via self-assembly at intracellular plasma membranes, and signal transduction interfaces between cytoskeleton- and membrane-bound components. At the structural level, GBP1 is a large 65 kiloDalton protein in which distinct N- and C-terminal domains are joined by a GTPase hinge region, whose nucleotide binding status regulates the function of GBP1's other domains (1, 2). At the mechanistic level, GBP1 self-assembles into tetramers in some contexts while heterodimerizing with diverse partners, such as F-actin or p62, in other contexts (3, 4). At the cellular level, GBP1 activation both restrains proliferation and protects against apoptosis in inflammatory contexts (5–7). Finally, at the phenomenological level, GBP1 appears to act as a shepherd of IFNγ-induced cellular responses, where GBP1 does not directly participate in the reaction to cytokines but instead attenuates the signal intensity thereof over time (8–10). In infection, these attributes of GBP1 cooperate to prevent proliferation of infected cells, stymy premature angiogenesis, and protect stroma from inflammation-induced apoptosis while coordinating cell-autonomous immunity functions including autophagocytosis (11–13). In homeostasis, GBP1 appears to participate in endosomal trafficking, cytoskeleton regulation, and autophagy (4, 14). However, in the setting of cancer, GBP1 may act as a double-edged sword, capable of restraining cancer cell proliferation in some contexts—such as breast and colorectal cancers—but also sufficient to anchor a potent chemotherapy treatment resistance (TXR) phenotype when driven by upstream oncogenes, inflammation, and/or the cellular response to the cytotoxic mechanisms of chemotherapy in ovarian cancer and glioblastoma (15–18).

## Structure of GBP1

**Figure 1:**
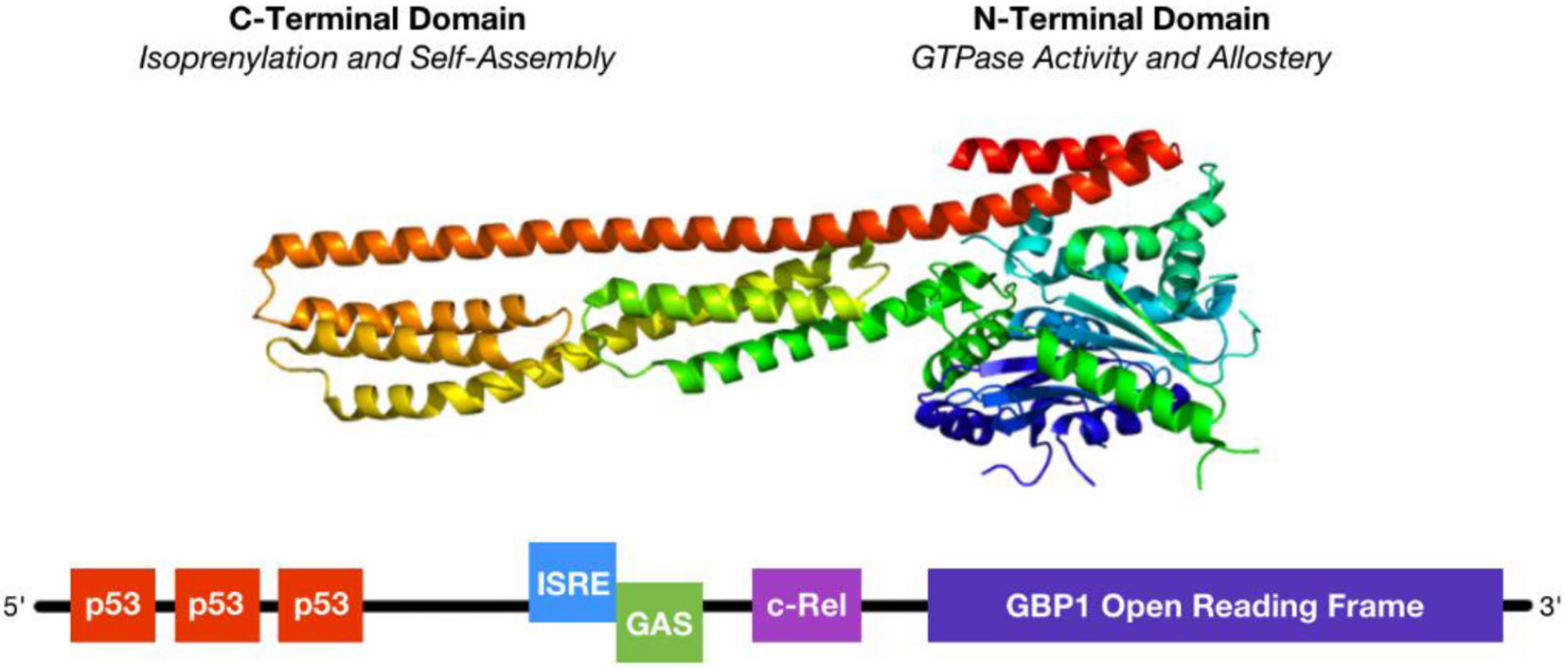
Structure and regulation of GBP1. **(Top)** GBP1 is a large, 65 kDa GTPase whose activity depends upon homodimerization or tetramerization. Self-assembly is regulated by GTP binding status, where GTP binding initiates activity and self-assembly and either GDP or GMP may bind to the GTPase site as competitive inhibitors. **(Bottom)** GBP1's promoter region is regulated by several distinct, but overlapping, transcription factor binding sites. First, 3 p53 responsive elements lay upstream and can be bound by multiple copies of p53 at a time. Second, the ISRE (interferon-stimulated response element) site can be occupied by IRF1, IRF9, or STAT1 singly or in dimers, similar to the downstream GAS (γ-interferon activation site), following stimulation with either IFNα or IFNγ. Third, the c-Rel site preferentially binds to the p50 sub-unit of NFκB, whose lack of a transactivation domain suggests functional heterodimerization with other transcription factors. Binding of multiple transcription factors yields additive activation.

The human form of GBP1 is a large, 65 kDa GTPase comprising a globular N-terminus made of jumbled α-helix and β-sheet domains connected to a long C-terminus of parallel α-helices (Figure 1). The N-terminus contains a nucleotide binding site, termed the Large GTPase (LG) domain, shared among other members of the dynamin superfamily which collectively regulate membrane trafficking, and an α4′ helix hinge region. The C-terminus contains a conserved CaaX isoprenylation site as well as α12 and α13 helices that engage in regulatory electrostatic interactions with the α4′ helix at the interface with the LG domain. Unlike the related GTPases Ras and Gα, GBP1 does not require a co-activating binding factor to exhibit enzymatic activity (19–21). Instead, GTP binding to the LG domain triggers conformational changes in the α4′ helix, which in turn pulls α12 and α13 into a more exposed configuration that enables homodimerization of GBP1 via interaction of the α12/13 domains on one monomer with the α4′ helix on the other monomer. Once dimerized with 2 bound GTP nucleotides, the LG domains of the GBP1 homodimer cleave the first phosphate group from GTP, at which point the freed γ-phosphate may be released from the complex or held in place by the β-phosphate still bound to the α-phosphate. However, GBP1 can also produce GMP directly from GTP, where release of a two phosphate groups suggests an alternative conformation in which GBP1's C-terminal α12/13 domains prevent dissociation of the GBP1 dimer and immediate release of the γ-phosphate. The N-terminal LG domain can also act to cleave the β-phosphate from the α-phosphate, after which one or two phosphate groups may be released upon dimer dissociation. At each enzymatic step, conformational shifts in the α12/13 and LG domains coordinate to prime the next cleavage reaction (1). GTP, GDP, and GMP all exhibit similar binding affinities for GBP1, where they compete for access to the binding site (22). Although phosphate group release has been observed from GBP1 dimers upon their dissociation, GBP1 dimers have also been shown to form a GTP or GDP nucleotide-bound tetramer from which GBP1 monomers may dissociate upon GMP product release (23). These results suggest that the GTP-to-GDP transition state favors GBP1 dimerization while GTP-to-GMP 2-step cleavage may be driven by the transient formation of GBP1 tetramers (3). While this is supported by the inability of monomeric or dimeric GBP1 to produce GMP directly from GDP, additional studies will be necessary to fully elucidate the molecular dynamics of nucleotide transition states and GBP1 self-assembly. Current data supports a model in which transient self-assembly of GBP1 is governed by local availability of GTP, GDP, or GMP, wherein only GTP stimulates enzymatic cleavage while GDP and GMP act as competitive inhibitors of further nucleotide cleavage.

**Figure 2:**
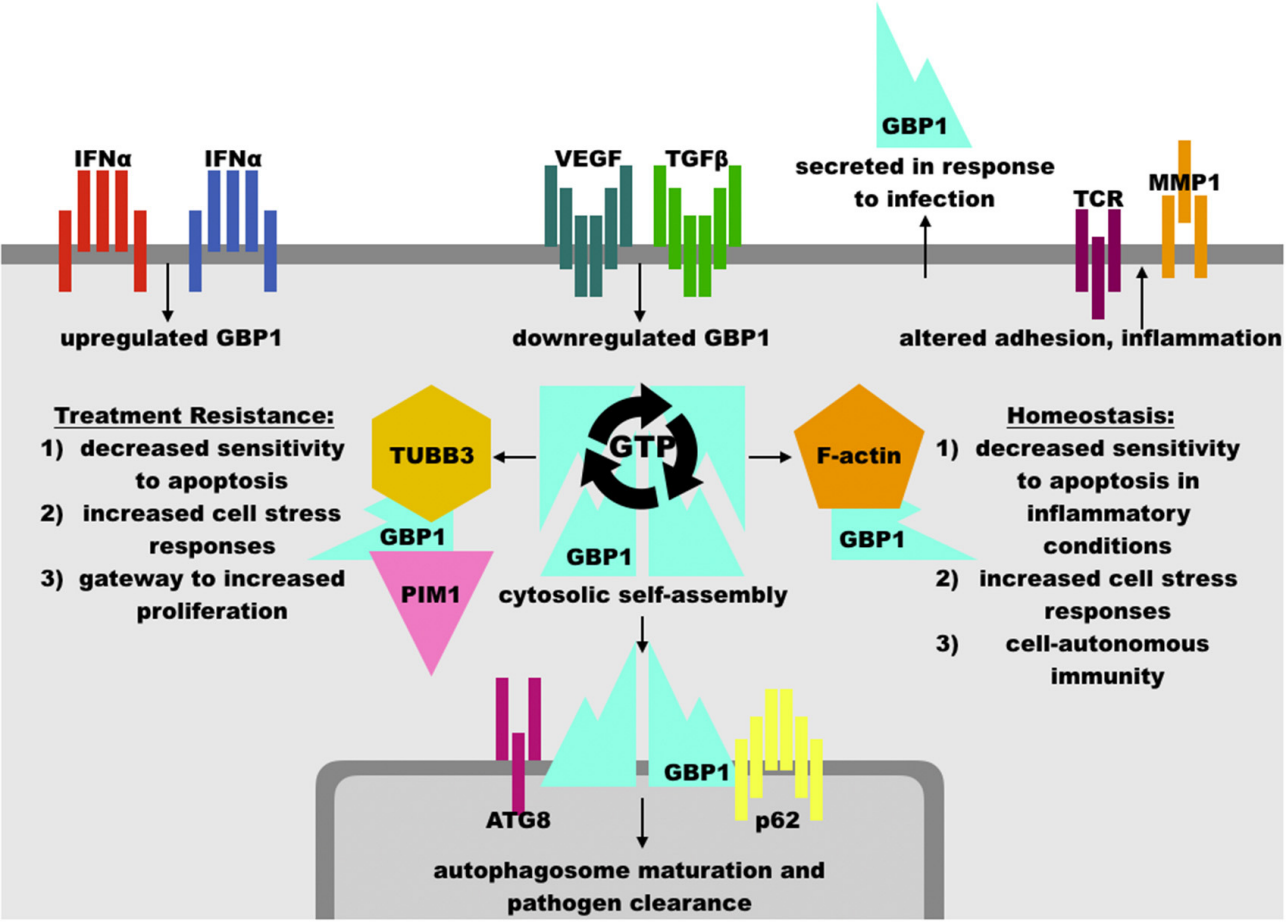
Overview of GBP1 function. GBP1 may be upregulated in response to interferons or other inflammatory cytokines, such as TNFα, and is downregulated by growth factors such as vascular endothelial growth factor (VEGF) and transforming growth factor β (TGFβ). GBP1 may localize cytosolically, at membrane surfaces, or extracellularly via secretion. GBP1 self-assembly and binding partner accessibility are regulated by its GTP binding status, where GTP favors assembly while GDP inhibits it. GBP1 has been found in complex with TUBB3 (β tubulin III), PIM1 (Pim-1 proto-oncogene, serine/threonine kinase), ATG8 (autophagy-related protein 8), p62 (sequestosome 1), F-actin monomers, and others, which facilitates its diverse cellular effects. These include an essential role in the clearance of intracellular pathogens, arrest of cell proliferation in inflammatory conditions, and protection of cells from inflammation-induced apoptosis. These activities may be hijacked by upstream oncogenic events to promote cancer cell survival against cytotoxic therapies, such as paclitaxel and radiation therapy. Other functions of GBP1, such as modulation of inflammatory state, its association with factors such as PIM1, and promotion of cell-cell adhesion dysregulation may serve to increase disease progression in diverse cancer sub-types, including ovarian, head-and-neck, and brain lesions. In human patients, mutations in GBP1 are associated with chronic viral infection, although mutation in GBP1 is not necessary for its involvement in treatment resistance.

**Figure 3:**
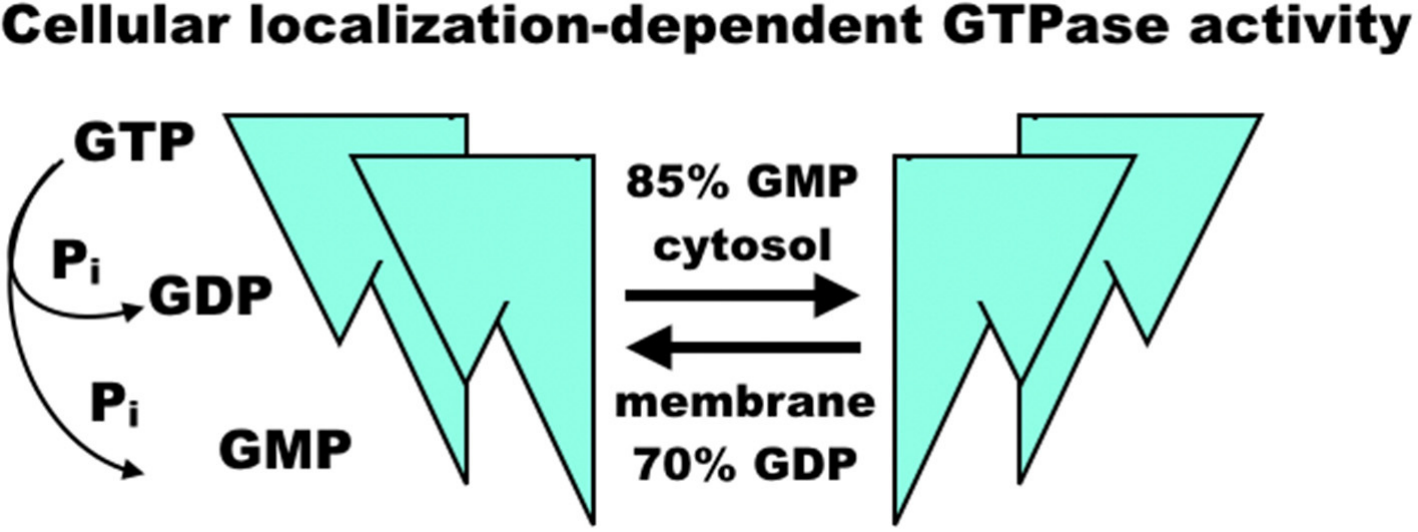
Model of GBP1 GTPase activity. GBP1 binding of GTP appears to trigger homodimerization and further self-assembly into homotetramers, fibrils, or larger molecular complexes with diverse binding partners regulating proliferation and inflammation. When membrane-bound, GBP1 primarily produces GDP, releasing a single phosphate, whereas cytosolic GBP1 preferentially produces GMP and a diphosphate group. The sequence of self-assembly states, functional relationship to diverse binding partners, and precise regulation of GTPase activity in each is, as yet, un-characterized.

The regulation of GBP1 function is complicated by its isoprenylation site at the C-terminal CaaX motif, where farnesylation is necessary for lipid membrane localization of GBP1 (24, 25) (Figure 2). GBP1 is a member of the dynamin superfamily, which drives vesicle cleavage through self-assembly at the membrane surface (26). It is not yet clear how GBP1 self-assembly plays a role in biology. In homeostatic conditions, GBP1 is expressed at very low levels, rising only in the presence of interferon signaling (27). Under inflammatory conditions, the pool of GBP1 proteins rapidly expands, which is essential in host immunity (described further below) as well as protection against apoptosis (7). From this, GBP1 appears to mediate some crucial lipid membrane interactions, although it is not yet clear whether these interactions resemble those undertaken by other members of the dynamin superfamily. GBP1's interactions with lipid membranes are further complicated not only by its dependence on GTP-mediated, farnesylated GBP1 activation and membrane binding, but also by mutual competition between self-assembly and membrane-binding states. GBP1 bound with GDP-aluminum fluoride or GMP cannot bind to lipid membranes; only the GTP-bound forms permit this interaction (28). Subsequent membrane dissociation of GBP1 is rapid as GTP is cleaved to GDP or GMP, which can then competitively inhibit re-association of GBP1 with lipid membranes. While farnesylation of the CaaX motif is a necessary prerequisite to GBP1 binding of lipid membranes (24), it does not appear to inhibit GBP1 self-assembly in the cytosol (3, 24). This does, however, alter GBP1 activity in that cytosolic GBP1 preferentially produces 2 GMPs from GTP (85% GMP to 15% GDP), while membrane-bound GBP1 is biased toward GDP production from GTP (70% GDP to 30% GMP) (22, 23) (Figure 3). In experimental conditions using purified farnesylated protein, GBP1 was found to self-assemble into round tubes with a diameter of 60 nm and a length between 60 and 200 nm in the presence of GTP whereas GDP resulted in fewer, smaller tubes 20 nm in diameter and 50 nm in length (29). The biological function of these self-assembled structures is, at present, unknown, although recent work has shown that tetramerization and its associated preferential production of GMP are allosterically linked to antiviral activity *in vitro* (30). That GBP1 also binds to globular F-actin and β-tubulin-III (described further below) has interesting implications for cell biology that warrants further investigation (4, 17), as do recent molecular dynamics simulations suggesting that self-assembly may be driven by dynamin-like large-scale rearrangements of alpha helices throughout GBP1's structure (31).

## Regulation of GBP1

Several lines of evidence show that GBP1 is a critical interferon-stimulated gene (ISG) unique in the speed and magnitude of its upregulation after type-I or -II interferon stimulation (27). Unlike other slower ISGs, GBP1 levels spike within an hour of type-I interferon exposure and continue to rise for up to 24 h, by which point slower-responding ISGs are also expressed (9, 10). A similar, but slower, rise in GBP1 is also seen with type-II interferon (27). GBP1 levels remain high with continued interferon stimulation but rapidly fall after withdrawal of interferon stimulus (32). While many ISGs, such as various cytokine receptors or chemokine ligands, directly mediate the cellular response to inflammation, GBP1's mechanism of action appears to be more indirect through the attenuation of overall ISG activity as an integrator of cell stress and inflammatory programs, as evidenced by its regulatory effects on the magnitude of interferon secretion, activation of autophagy, and decreased sensitivity to apoptosis, which may be a protective mechanism in epithelial and endothelial cells to prevent cell death upon initiation of inflammation (6–9). Except for initial results showing GBP1's diverse putative binding partners, from p62 to globular F-actin (4, 33), the exact nature and extent of GBP1's downstream binding partners or the influence of GBP1's nucleotide-, membrane trafficking,- or self-assembly-states thereupon remains to be elucidated. Overall, this leads to the phenomenological model of GBP1 activity in which GBP1 facilitates the early cellular inflammatory response via activation of cytoskeletal remodeling and endosome trafficking while also serving to prevent runaway positive feedback from mounting an out-of-control inflammatory response or premature cell differentiation (11, 34).

The GBP1 promoter region contains three distinct inflammation-associated sequences (Figure 1): an interferon-stimulated response element (ISRE), a γ-interferon activation site (GAS), and a c-Rel binding site (9, 10, 27, 32). Each of these sequences is associated with distinct, but overlapping, inflammatory pathways. Type-I interferons, such as interferon-α (IFNα) stimulate GBP1 transcription by activating interferon-stimulated gene factor 3 [ISGF3, also known as interferon-responsive factor 9 (IRF9)] at the ISRE site or by IFNα activation factor [AAF, a homodimer of signal transducer and activator of transcription 1 (STAT1)] at the GAS site, which in turn is more potently activated by type-II interferon, such as interferon-γ (IFNγ), but acts via GAGA-associated factor (GAF), IRF1, or IRF9. IFNα- and IFNγ-stimulated signaling is not mutually exclusive; activation at the ISRE site via either IFNα or IFNγ or at the GAS site via IFNγ can stimulate GBP1 transcription, but for this effect the ISRE site is strictly necessary as its genetic knock-out blocks the rise in GBP1 after interferon-α or -γ stimulation (32). GBP1's promoter contains another ISG binding site as well: c-Rel, to which the p50, instead of the more commonly-used p65, subunit of NFκB binds in response to tumor necrosis factor α (TNFα) or interleukin-β1 (ILβ1) signaling (9, 10). However, p50 lacks a transactivation domain and can only promote GBP1 transcription with a heterodimerized binding partner, such as CCAAT-enhancer binding protein β (C/EBPβ) or B-cell lymphoma 3-encoded protein (BCL3) (35). Transcription factor activity at the c-Rel site is cooperative with the ISRE and GAS sites and is necessary for full activation of GBP1 transcription, as shown by its knockout diminishing—but not eliminating—GBP1 production after IFN-mediated activation (32). Interestingly, both the ISRE/GAS and ISRE/c-Rel pairings are found in the promoter sequences of other important ISGs, such as CD1b, CD69, and regulated on activation, normal T-cell expressed and secreted (RANTES, also known as CCL5), suggesting that this is a conserved regulatory motif in many ISGs that may be used to coordinate cellular activation states with diminished apoptotic sensitivity needed for effective cell-autonomous immunity and function in a highly-inflamed microenvironment (9). Thus, stimulatory cytokines may initially promote GBP1 synthesis on a rapid time-scale to coordinate cellular changes for slower-responding ISGs to then “fine-tune” inflammation, providing a parsimonious activation mechanism for inflammatory reactions as well as the means of regulating them. Furthermore, the heterogeneous binding partners of p50 at the c-Rel site are implicated in driving differential inflammatory outcomes; for example, C/EBPβ activity is associated more with the immunosuppressive phenotype in activated myeloid cells (35), where GBP1 is also highly expressed after cytokine stimulation. Additional elucidation of how transcription factor heterodimers drive differential expression patterns of ISGs will substantially further our understanding of the early inflammatory response process.

GBP1 is also regulated via the p53 and Yin Yang 1 (YY1) transcription factors. GBP1 has three p53-binding sites further upstream in its promoter that act cooperatively with downstream ISG sites (36). Its YY1 site is activated via p38-mitogen associated protein kinase (p38-MAPK) activity (18). Both p53 and p38-MAPK are activated by cellular stressors, such as DNA damage, TNFα, IL1α, Fas ligand (FASL), and transforming growth factor β (TGFβ) and act to coordinate growth cycle arrest, decreased sensitivity to apoptotic stimuli, and upregulation of various cell stressor response factors, such as heat shock proteins, Jun nuclear kinases, and the Ras family of small GTPases (37). These factors imply that GBP1 may play an essential role in the cellular response to various genotoxic, cytotoxic, and inflammatory signal stressors, promoting resistance to apoptosis while organizing a wide variety of stressor response effectors that enable continued cell survival in harsh conditions.

## GBP1 in Inflammation

As noted above, interferon and other cytokine stimulation potently activates GBP1 activity. Given that many inflammatory stimuli increase the sensitivity of receiving cells to apoptosis, GBP1's rapid induction and subsequent inhibition of cellular proliferation and apoptosis after cytokine exposure appears to act as a mechanism to increase cell survival in the immediate aftermath of an inflammatory event. In endothelial cells and endothelial progenitors, IFN exposure induces a fast spike in GBP1 levels, whose C-terminal is sufficient to restrain cellular proliferation in endothelial and epithelial cells via an as-yet-undefined mechanism and whose N-terminal GTPase function is necessary for the inhibition of matrix metalloproteinase 1 (MMP1), which, in combination with upregulated integrin α4, reduces cellular spreading and proliferation capacity independently of GBP1's C-terminal effects (6, 10, 38) (Figure 2). Increased integrin α4 does, however, facilitate leukocyte adhesion at GBP1-high endothelial cells, suggestive of a role in facilitating immune mobilization at sites of inflammation (39). Multiple studies knocking-out or over-expressing GBP1 via transgenic or synthetic constructs have shown that GBP1 is necessary and sufficient for inhibition of proliferation after inflammatory cytokine exposure in endothelial cells (6, 14). These effects are antagonized by increasing exposure to fibroblast growth factor β (βFGF) and vascular endothelial growth factor (VEGF), which also decrease GBP1 expression in human umbilical vein endothelial cells (HUVECs) (32) and in a dose-dependent manner in 3D engineered tissue constructs where greater expression of GBP1 correlated with decreased angiogenesis (40).

High GBP1 expression after interferon exposure has also been found in leukocytes and intestinal epithelial cells (14, 41). Although GBP1 is essential for autonomous host defense against intracellular pathogens in myeloid cells (expanded upon below), its direct role in myeloid cell proliferation has yet to be elucidated, although initial results suggest that GBP1 activity is associated with a more immunosuppressive “M2” myeloid phenotype (42). GBP1 is highly expressed in both CD4 and CD8 T-cells after interferon stimulation, where its levels continue to rise until 24 h after stimulation (3). Genetic knock-down of GBP1 in T-cells leads to significantly higher interleukin-2 (IL2) secretion, greater T-cell receptor and CD3 signaling, and increased CD69 and CD45 expression, both markers of cell activation, although downstream ERK phosphorylation was also increased (14), suggestive of greater cell stress response. Thus, GBP1 may act to preserve cell viability and moderate the reaction to interferon signaling to prevent both a crash in T-cell population while also constraining positive paracrine or autocrine feedback in pro-inflammatory signaling. These effects are mediated through broad interaction of GBP1 with regulatory cytoskeletal proteins, such as plastin-2 (LCP1), β-2 spectrin, and myosin-associated factors (14). Interestingly, separate research has shown that GBP1 inhibits the formation of filamentous actin via oligomerization-mediated preferential binding and stabilization of globular F-actin (4), which, given the necessity of extensive actin remodeling to form an effective immunological synapse in T-cell activation or during leukocyte chemotaxis (14), suggests that GBP1 could play a role in slowing the adoption of pro-activation effectors into the cytoskeleton. Intriguingly, GBP1 levels are associated with an activated T helper 1-like phenotype in T-cells interacting with inflamed intestinal epithelial cells (43), showing that GBP1 does not hinder longer-term T-cell function. GBP1 also plays a role in intestinal epithelia in chronic inflammatory conditions, co-localizing at membrane-adjacent actin sites with coxsackie- and adeno-virus receptors where, similar to its role in endothelial cells, GBP1 activity is correlated with increased survival and decreased proliferation upon inflammatory stimulus (44). *2012 Capaldo Nusrat* showed that GBP1's blockade of intestinal epithelial cell proliferation is regulated independently of canonical glycogen synthase kinase 3 β (GSK3β)-mediated inhibition of pro-mitogenic β-catenin and T-cell factor signaling (41). That GBP1 expression correlates with both inflammatory bowel disorder and decreased growth of colorectal carcinomas suggests that GBP1 acts in a similar manner in epithelial cells as it does in endothelial cells to decrease proliferation and inflammation-induced apoptosis (40, 41, 43, 44), which has profound implications for mucosal immunity and treatment.

In sum, GBP1 acts to inhibit proliferation and cellular sensitivity to apoptosis in response to inflammatory stimuli via regulation of the cytoskeleton and associated signaling scaffolds on short time scales.

## GBP1 in Infection

GBP1 plays an essential role in cell-autonomous immunity, in which infected cells rely upon maturation of phagosomes/infected vacuoles into autophagosomes to destroy invading pathogens or, if this cannot clear the infection, pyroptosis to trigger increased immunity at the site of infection. In line with these findings, it has been found that GBP1 rapidly localizes to phagosomal membranes in monocytes infected with *Listeria, Chlamydia*, or myriad viruses, where it is necessary but not sufficient to promote maturation of autophagosomes, participates in inflammasome formation and pathogen-associated molecular pattern sensing, and is needed for IFNγ to exert anti-viral effects in *in vitro* experiments with primary human cells and *in vivo* in mice (13, 45–49). Like other members of the dynamin family of large GTPases, which are also involved in cell-autonomous immunity, GBP1 appears to cooperate with numerous membrane regulatory elements, such as phagocyte oxidase and p62/sequestosome 1, which promote effective autophagy and infection control (33). This accords with GBP1's role in the promotion of cell stress responses via ERK pathways, coupling regulation of membrane dynamics (including mitochondrial) to metabolic and inflammatory function (14, 41, 42). Indeed, knock-out experiments have shown that human cell lines lacking GBP1 fail to resolve intracellular pathogen infections while epidemiological evidence in humans heavily implicates polymorphisms in GBP1 with chronic active Epstein-Barr virus infection, ulceroglandular tularemia, and elevated GBP1 in the cerebrospinal fluid of patients with bacterial meningitis (33, 50). These lines of evidence suggest that GBP1 not only plays a role in cell responses to interferon or other cytokine stimuli but is also essential for intracellular defense in a variety of settings. Whether these defensive functions also include oxidative or other cellular damage response pathways remains to be seen.

## GBP1 in Cancer

Cancer emerges from a complex interplay of mutational events and cell state transitions that are accompanied by interferon-mediated inflammation and cytoskeletal reorganization which may involve GBP1 in the oncogenic process. The effects of GBP1 in cancer appear to be highly context-dependent, however, as its upregulation is associated with decreased progression in some cancer types, such as breast and colorectal cancer (51–53), but is linked with increased progression, metastasis, and treatment resistance in other cancer types, such as ovarian cancer and glioblastoma (17, 54). Although GBP1 may exert these effects in a cancer-intrinsic manner by acting within cancer cells or via a cancer-extrinsic mechanism through microenvironment and immune activity, current literature does not yet distinguish clearly between these contributions and additional investigation is urgently needed.

In breast, colorectal, and skin cancers, transcriptional and immunohistochemical profiling of patient samples has revealed that high GBP1 signatures are favorable prognostic indicators (15, 51–53, 55, 56) associated with decreased disease progression and greater overall survival. These findings may be driven by GBP1's anti-proliferative activity in an inflammatory context, as seen in inhibition of endothelial and epithelial proliferation after cytokine stimulation (6, 7). The degree to which this is true in the complex setting of the tumor microenvironment is, at present, unknown and further studies are needed to delineate the lineage-specific activity of GBP1 in the tumorigenic process. And while GBP1 may act to restrain cancer cell proliferation in these cancers, it may also act as a mediator of treatment resistance. In a study of human breast cancer samples, Fekete and Györffy (57) found that GBP1 expression strongly correlated with paclitaxel treatment failure across all molecular subtypes of breast cancer, although this is in contradiction to findings from Tipton et al. (58) using genetic manipulation of GBP1 in human cell lines that found GBP1 over-expression did not induce paclitaxel resistance. Similar complexity has been found in colorectal cancer, where loss of GBP1-mediated apoptotic responsiveness to interferon signaling is associated with co-regulation of genes involved in immunosuppression, which facilitates disease progression and portends worsened prognosis (59).

In ovarian and head-and-neck cancers, however, GBP1 has been consistently associated with disease progression and the development of treatment resistance (17, 60). This trend was first noted in profiling of human ovarian cancer cell lines, which found that high GBP1 expression strongly correlated with paclitaxel resistance and that its knock-out restored treatment sensitivity (60, 61). These findings have been borne out in examination of patient samples, where, in a separate paper, Wadi et al. (62) replicated previous findings of GBP1's sufficiency to protect against paclitaxel-mediated apoptosis and also found high GBP1 expression in 17% of pre-treatment ovarian cancer patients. This figure was increased to 80% of ovarian cancer patients after treatment with paclitaxel or docetaxel and follow-up study found GBP1 expression was a significant correlate of decreased progression-free survival (62). Similar results have been found in squamous head-and-neck cancers, where GBP1 expression correlates with disease stage and lymph node metastasis in patient samples (63, 64). GBP1 expression has also been correlated with metastasis in both lung and breast cancers as well, where study of human lung cancer explants and brain-metastasizing breast cancer cells showed decreased migration *in vitro* upon GBP1 blockade and GBP1 expression correlated with disease progression in patients (54, 65). Intriguingly, in breast cancer metastasis, it was found that GBP1 expression was induced in breast cancer cells by T-lymphocyte activity, showing that cancer-extrinsic processes may influence GBP1's context and function (54). One aspect of this differential activity may be influenced by heterogeneous binding partners, such as the findings that GBP1 interacts directly with proto-oncogene serine/threonine kinase 1 (PIM1), a marker of disease progression and treatment resistance in ovarian and other cancers (66, 67). In Tipton et al. (58), it was shown that the differential activity of GBP1 in paclitaxel sensitivity between breast and ovarian cancer cell lines was mediated by interaction with different isoforms of PIM1, wherein a 44 kDa PIM1 isoform was associated with unaltered paclitaxel sensitivity in breast cancer cells while a related 33 kDa PIM1 isoform was associated with induction of paclitaxel resistance in ovarian cancer cells. These results underline that additional study of GBP1's role in tumorigenesis is not only compelling, but will also require careful attention to the context in which it acts, including cancer type, treatment history, and model system. Although all results above are from patient samples or human cell lines, it must be noted that human GBP1 is more homologous to murine GBP2, a closely related GTPase (68), that bears special examination when using syngeneic murine cell lines for further study of GBP biology and cancer progression.

## Discussion

GBP1 is a unique large GTPase governing cellular responses to infection, inflammation, and environmental stressors that, in some contexts, can be hijacked by upstream oncogenic events to induce treatment resistance and tumor progression in cancer. Unlike other interferon-inducible genes that take several hours to respond to inflammation, constitutively low levels of GBP1 are rapidly upregulated in response to IFNγ, IFNα, TNFα, or IL-1α/β within an hour and continue rising up to 24 h. GBP1 does not appear to directly participate in inflammatory signaling, instead it modulates the magnitude of the inflammatory response in T-cells, myeloid cells, endothelial cells, and intestinal epithelial cells (IECs), where GBP1 activity results in reduced inflammatory cytokine secretion as well as reduced sensitivity to inflammation-mediated apoptosis. These effects are linked to GBP1's profound anti-proliferative effects in endothelial cells and IECs, where the non-GTPase C-terminal domain suppresses cellular proliferation independently of its farnesylation status and membrane localization. Although the exact molecular linkages aren't yet known, GBP1 activity is also associated with increased c-Jun and ERK pathway activation, implying a role in the activation of cell stress response pathways and unifying GBP1's role as a potent inhibitor of runaway positive feedback in inflammatory signaling and thus reducing the cell death that would otherwise accompany such signaling.

GBP1 has been observed in multiple cellular compartments with a broad array of binding partners, including cytosolic self-assembly of homotetramers into filaments, participation in F-actin globular assemblages, homodimeric association with membrane surfaces, heterodimeric signaling, and monomeric extracellular secretion. The binding preferences of GBP1 appear to be regulated by its nucleotide binding status, wherein GTP binding enables homo-dimer or -tetramerization and subsequent cleavage of GTP to GMP whereas binding of either GDP or GMP to GBP1's GTPase site competitively inhibits self-assembly. The exact relationships between GBP1 binding partners and its diverse cellular effects are incompletely characterized, although it has been shown that GBP1 interaction with p62 at the endosome surface is necessary for maturation into autophagosomes in the clearance of intracellular infections in monocytes and that GBP1 interacts with the actin cytoskeleton in reducing the response to inflammatory stimuli in T-cells. GBP1 has also been caught binding to TUBB3, an alternative microtubule subunit upregulated in taxane-resistant cancer cells, and PIM1, a potent promoter of cellular proliferation, in the formation of a cytoskeletal gateway of multi-drug treatment resistance (TXR) in ovarian, head-and-neck, breast, and brain cancers, implicating it as a key target in the fight against TXR^+^ disease and its associated mortality.

While GBP1 is anti-proliferative in normal cells, acting to restrain premature angiogenesis or IEC proliferation in inflammatory conditions, this effect is highly context-dependent in cancer. Whereas, the growth of some cancers, such as colorectal carcinoma and breast cancer, may be restrained by GBP1 activity, GBP1's pro-survival effects can also be hijacked by common oncogenic mutations, such as EGFRvIII, to supercharge its anti-apoptotic effects while the activity of other cooperating factors, such as PIM1 or p38-MAPK, drown out any latent GBP1 anti-proliferative activity and result in an aggressive, TXR^+^ cancer phenotype. Indeed, GBP1 protein levels are negative prognostic indicators in ovarian and head-and-neck cancer patient samples and are implicated in the progression of lung, brain, and other cancers as well. Genetic manipulation of GBP1 levels have shown that GBP1 activity is both necessary and sufficient to mediate cell survival even in the face of increasing doses of taxanes, platinum agents, and radio-therapy both *in vitro* and *in vivo*. Furthermore, preliminary experiments with small molecule-mediated inhibition of GBP1's association with PIM1 have demonstrated restoration of treatment sensitivity and reduction in ovarian cancer growth, showing that GBP1 is an excellent molecular target for combating TXR in tumor progression. Success in such therapies would not only restrain tumor growth, but also increase the cytotoxic efficacy of taxanes and other cancer therapeutics, enabling lower doses to exert greater effects and rescuing patients with otherwise-refractory disease.

Though GBPs have been characterized for over three decades, there remain many unanswered questions surrounding GBP1 biology. Chief among these is the precise nature of the relationship between GBP1 cellular localization, its binding partners (including itself), and its cellular effects in health and disease in various cell types. It will be of particular interest to determine whether GBP1's effects are cell type-specific or instead more universally used to protect against inflammatory insult and, furthermore, how these effects differ by nucleotide binding status and post-translational modification. The non-proliferative, anti-apoptotic, and inflammation-attenuating activities associated with GBP1 activity in homeostasis and TXR in cancer also closely resemble the emerging hallmarks of cellular senescence, which is implicated in tumorigenesis and the regulation of inflammation and wound healing. Elucidation of these and other questions, such as the identity of other factors cooperating with GBP1 in membrane trafficking, TXR, and cell stress response, will yield rich new insights into the regulation of inflammation, cell survival in the face of cytotoxic stressors, the progression of myriad forms of cancer, and the therapeutic means to alter GBP1-related activities to improve control of infection, cancer, inflammation, and senescence.

## Author Contributions

SM conceived the idea of this review article. AH, DT, and SM contributed in writing this manuscript.

### Conflict of Interest

The authors declare that the research was conducted in the absence of any commercial or financial relationships that could be construed as a potential conflict of interest.

## Abbreviations

GBP1: guanylate-binding protein 1, a large GTPase involved in cell survival and proliferation
GTPase: an enzyme that modifies the phosphate groups on guanosine-5′-triphosphate
IFNγ: interferon γ, a potent anti-viral and pro-inflammatory cytokine
TXR: treatment resistance
LG: large GTPase domain of GBP1
CaaX: amino acid isoprenylation site
GTP: guanosine-5′-triphosphate
GMP: guanosine-5′-monophosphoate
GDP: guanosine-5′-diphosphate
ISG: interferon-stimulated gene
ISRE: interferon-stimulated response element
GAS: γ-interferon activation site
IFNα: interferon alpha, an inflammatory cytokine
ISGF3: interferon-stimulated gene factor 3
IRF9: interferon-responsive factor 9, another name for ISGF3
IRF1: interferon-responsive factor 1
NFκB: nuclear factor κB, a transcription factor involved in regulation of inflammation
TNFα: tumor necrosis factor α, a common pro-inflammatory mediator
ILβ1: interleukin β1, a common inflammatory cytokine
C/EBPβ: CCAAT-enhancer binding protein β, a transcription factor
BCL3: B-cell lymphoma 3-encoded protein
CD1b: a transmembrane glycoprotein involved in presentation of lipid antigens
CD69: a marker of T-cell activation
RANTES: regulated on activation, normal T-cell expressed and secreted, also known as CCL5
YY1: Yin Yang 1 transcription factor
FASL: Fas ligand, an apoptotic signal
MMP1: matrix metalloproteinase 1
βFGF: fibroblast growth factor β
VEGF: vascular endothelial growth factor
M2: putative immunosuppressive myeloid cell state
ERK: extracellular signal-regulated kinase, a regulator of cell receptivity to exogenous signals
LCP1: plastin-2, a cytoskeletal binding protein
GSK3β: glycogen synthase kinase 3β, involved in glucose storage
MOA: mechanism of action
TUBB3: β tubulin III, an alternative microtubule subunit
ABC: ATP-binding cassette, a motif common to energy-consuming proteins
MDR-1: multidrug resistance 1, a small molecule efflux pump
PIM1: protoviral integration site 1, also known as proto-oncogene serine/threonine kinase 1
CDKN1A: cyclin-dependent kinase inhibitor 1a, a regulator of cell cycle progression
EGFRvIII: a common oncogenic mutation in the epidermal growth factor receptor
P38-MAPK: a mitogenic sensing and regulatory pathway
TP53: a common oncogene that dysregulates cell cycle progression
STAT1: signal transducer and activator of transcription 1.
